# Modulation of angiogenic transcription factor ERG via miR-6756 and miR-3190

**DOI:** 10.1371/journal.pone.0358739

**Published:** 2026-09-25

**Authors:** Sidra Mumtaz, Muhammad Usman Rashid, Safir Ullah Khan, Naila Malkani

**Affiliations:** 1 Department of Zoology, GC University, Lahore, Pakistan; 2 Department of Basic Sciences Research, Shaukat Khanum Memorial Cancer Hospital and Research Centre (SKMCH & RC), Lahore, Pakistan; 3 Moores Cancer Center, University of California, San Diego, United States of America; Astellas, UNITED STATES OF AMERICA

## Abstract

Angiogenesis plays a crucial role in cancer metastasis as tumors need to establish an increased blood supply to satisfy the demand for oxygen and nutrients. Numerous genes regulate this process, and various mechanisms including microRNAs regulate the expression of these genes. In the current study, we hypothesized that miR-6756 and miR-3190 regulate angiogenic mediators such as transcription factor ETS-related gene (ERG). Detailed bioinformatics analyses were performed to locate binding sites of miR-6756 and miR-3190 on ERG mRNA. miR-6756 and miR-3190 were cloned and overexpressed in primary endothelial cells to analyze ERG expression. To evaluate ERG activity reporter assays were performed in response to miR-6756 and miR-3190 over-expression. The effects of miRs on angiogenesis were measured by cell migration rate using the scratch assay. The expression of angiogenic targets of ERG was analyzed through qPCR with and without miR-6756 and miR-3190 overexpression. Lastly, VE-cadherin; an endothelial-specific ERG target was analyzed after miRs overexpression. Bioinformatics analyses showed that both miR-6756 and miR-3190 each have three binding sites on the mRNA of ERG. ERG expression at both mRNA (*p < 0.001*) and the protein level (*p < 0.001*) was significantly reduced after miR-6756 and miR-3190 overexpression. Also, the transcriptional activity of ERG significantly reduced (p < 0.05) in response to miRs overexpression. Cell migration rate (*p < 0.001*) and ERG angiogenic target genes were also decreased significantly (*p < 0.001*). VE-cadherin also showed significantly reduced expression and activity after miR-6756 and miR-3190 over-expression. This study indicates that miR-6756 and miR-3190 can regulate ERG expression and its target genes.

## Introduction

Angiogenesis is defined as the process by which new blood vessels are formed from pre-existing ones. Usually, it is strictly regulated and occurs during embryonic development, wound repair, folliculogenesis, and the menstruation cycle [1]. However, in some pathological conditions like arthritis, blindness, psoriasis, and cancer, excessive angiogenesis is also observed [2]. It is known that it plays a crucial role in cancer metastasis as tumors need to establish an increased blood supply to satisfy their high demand for oxygen and nutrients caused by their elevated metabolism [3]. When growing cancer develops a vascular network, the latter not only supplies nutrients for the tumor to grow but also offers an escape route for the tumor cells to enter circulation. Generally, it can be stated that the vascular density within and around the tumor, correlates with the risk for metastasis [4].

At the molecular level, angiogenesis results from an imbalance between proangiogenic and anti-angiogenic factors, with VEGF (vascular endothelial growth factor) or FGF (fibroblast growth factor) belonging to the first group; and angiostatin or endostatin belonging to the second category [5]. Proangiogenic factors like VEGF activate endothelial cell tyrosine kinases and subsequent downstream intracellular signaling through mitogen-activated protein kinase and phosphatidylinositol-3-kinase- (PI3K)/Akt/mTOR pathways leading to blood vessel formation [6]. The regulation of these factors is achieved by many different transcription factors such as the hypoxia-inducible factor HIF1α [7] or AP-1 [8]. But other endothelial cell-specific transcription factors play additional important roles in the whole process of angiogenesis. One of them is ERG (Ets-Related Gene), which is a member of the E-26 transformation specific (ETS) family of transcription factors. It is mapped on chromosome 21 (21q22.2) and encodes a 54 kDa (kilo Dalton) protein (P11308) [9]. ETS proteins are nuclear DNA-binding phosphoproteins that act as activators or repressors of transcription. ERG is primarily expressed in endothelial cells (ECs) [10], particularly in the blood vessels surrounding the neural tube [11], heart vasculature, and the pre-cartilage. It is involved in regulating several target genes and pathways needed for endothelial cell proliferation, survival, homeostasis, and barrier function [12]. Several model systems have established the fundamental role of ERG in angiogenesis, vascular development and stability [13]. ERG drives the expression of adhesion molecules like ICAM-2 [14] or junctional molecules such as VE-cadherin (VE-cad) [15], and is required for the stability of endothelial junctions [16]. Its long-term inhibition causes endothelial apoptosis [15]. ERG has also been implicated in the transcription of VEGF-R1 and 2 (VEGF receptor 1 and 2) [17,18], suggesting its role in regulating pro-angiogenic pathways directly. Owing to this significant role of ERG in angiogenesis, its expression must be tightly controlled in angiogenesis related diseases.

ERG expression is regulated in the cell through different mechanisms like post-translational modifications, alternative promoters, autoinhibition, and RNA-based regulation [19]. The latter occurs predominantly via microRNAs (miRNA), which control expression levels of their target genes either by triggering mRNA degradation or by inhibiting the translation of the target mRNAs. miRNAs are short single-strand RNAs with 19–25 nucleotides, which exhibit partial homology to sequences in their target mRNAs [20]. Several reports indicated that miRNAs have the potential to modulate angiogenesis [21–23] and might be interesting therapeutic agents in angiogenesis related diseases. Based on that, we intended to investigate candidate miRNAs predicted by various miRNA databases to act as potential modulators of ERG expression. This has led us to investigate miR-6756 and miR-3190 as possible regulators of ERG levels. ERG expression and its possible consequences on angiogenesis have been determined in response to miRs.

## Materials and methods

### Bioinformatics analysis for miRNA site prediction

A thorough bioinformatics analysis was performed to predict miR-6756 and miR-3190 binding sites on ERG mRNA. An online tool, miRDB (http://www.mirdb.org/cgi-bin/search_custom.cgi), was applied for this purpose.

### Cell culture

Human umbilical vein endothelial cells (HUVECs) were isolated from fresh umbilical cords under sterile conditions [24]. Cells were cultured in M199 (Medium 199) supplemented with penicillin (1000 units/mL), streptomycin (1000 μg/mL), 10% fetal bovine serum (FBS), 1mM L-glutamine and heparin plus ECGS (Endothelial cell growth supplement) with 5% CO_2_ at 37 °C. All assays on HUVECs were performed at passage 3 (P3). HEK 293 (Human embryonic kidney) cells were grown in DMEM (Dulbecco’s modified MEM medium) with 10% FBS and penicillin (1000 units/mL), streptomycin (1000 μg/mL).

### Expression plasmid and transfections

For constructing expression plasmids of miR-6756 and miR-3190, the precursor sequence harboring the mature sequence of miR-6756 (AGGGUGGGGCUGGAGGUGGGGCU) and miR-3190 (UGUGGAAGGUAGACGGCCAGAGA) were cloned into pEGFP-C1 vector (Clontech, 6084−1). A 300 bp fragment (244 bp upstream and 220 bp downstream of precursor sequences was PCR amplified from human genomic DNA using the following primers having restriction sites for HindIII and BamHI (Table 1).

**Table 1:** Primers for cloning miR-6756 and miR-3190.

miRNAs	Cloning Primers
hsa-miR-6756-5p	5’-TCGA**AAGCTT**GGTCAGTCTATTTAGATCAA- 3’ HindIII 5’-AGCT**GGATCC**GTAAGCAGAGAGTCAGGTTA- 3’ BamH1
hsa-miR-3190-3p	5’-TCGA**AAGCTT**ACTCCAGGTGCTGCTCCG- 3’ HindIII 5’-AGCT**GGATCC**TCTCTGGCCTTCTGGTCTCT- 3’ BamH1

These fragments were inserted downstream of eGFP in the multiple cloning sites of pEGFP-C1. For the ERG overexpression, a plasmid containing the ERG coding region was used as described [25].

HUVECs were transfected with these plasmids using electroporation [26].

### Luciferase reporter assay

Luciferase reporter gene assays were performed to assess the transcription activity of ERG as a result of miRNAs binding. The cells were seeded in 24 well plates and transfected with a reporter plasmid, beta-galactosidase, and gene of interest in triplicates. A constitutively expressing β-galactosidase construct was used for normalization in each sample. Reporter assays were performed in HEK 293 cells by transfecting appropriate reporter constructs and controls using Turbofect (Thermofisher, Cat. Nr. R0531). The normalized activity was measured by dividing luminescence values by respective β-galactosidase values [27].

In addition, to assess the role of ERG in the regulation of VE-cad expression, ERG DNA-binding deficient mutants were used. The W235R mutant has a tryptophan to arginine substitution at position 235 in the ETS domain that binds to DNA, while the RRAA mutant has two conserved arginine residues substituted by alanine, making it unable to bind to DNA. Functional assays were performed using these mutants to evaluate the ability of ERG to regulate VE-cad transcription.

### Quantitative real-time PCR

To detect the expression of miRNAs, ERG, and its downstream target genes (VE-cad, VEGF, FZD4, EGFL7) quantitative PCR (qRT-PCR) was performed using a StepOne plus Real-time PCR system (Applied Biosystems). HUVECs were transfected with appropriate plasmids and after 48hrs, cells were harvested and total RNA was isolated with TRIzol reagent [28]. RNA was transcribed into cDNA using the RevertAid cDNA synthesis kit (ThermoFisher Scientific; Cat: K1632) according to the manufacturer’s protocol. The obtained cDNA was used for qPCR using a SYBR green master mix (ThermoFisher Scientific; Cat: 4309155). All PCR reactions were performed in triplicates using appropriate controls. The data was analyzed using LinRegPCR software [29] and the baseline fluorescence, as well as the PCR efficiency of each amplicon, were calculated. The ΔΔCt-method was used to calculate changes related to controls [30]. For data normalization, U6 and GAPDH were used.

### Western blotting

HUVECs were transfected with miRs and ERG and the expression of the downstream target was analyzed at the protein level by Western blotting. 72 hours after the transfection, cells were lysed in RIPA (0.1M KH_2_PO_4_+0.1% TritonX100 pH 7.8) buffer on ice followed by centrifugation at 14,000 rpm at 4 ºC for 15 minutes. The total protein concentration of each sample was measured by Bradford assay and equal amounts of total protein from each sample were loaded on SDS-PAGE, followed by blotting and detection. Appropriate primary and secondary antibodies were used at the final dilution of 1:1000 and 1:10,000 respectively. The blots were visualized by enhanced chemiluminescence and quantified by ImageJ [31]. β-actin was used as a loading control.

### Scratch assay

The scratch assay was performed to analyze cell migration. HUVECs were transfected with miR-6756 and miR-3190, inoculated in 6-well plates, and grown until 90% confluent. Control cells were transfected with a pEGFP-C1 vector to rule out differences in cell treatment. Using a sterile P20 micropipette tip a scratch was made in the monolayer of transfected and untransfected cells. The scratch closure was observed at the interval of 4 hours. Cell migration was evaluated by measuring the difference in the cell free surface of the scratch at various time intervals using ImageJ [31]. Results were expressed as the percentage of scratch closure.

### Statistical analyses

Data were expressed as mean ± SD from three independent experiments and statistical significance was set at *p* < 0.05. The data were analyzed using GraphPad PrismV5.01.

## Results

### ERG mRNA is the predicted target for miR-6756 and miR-3190

**Fig 1:**
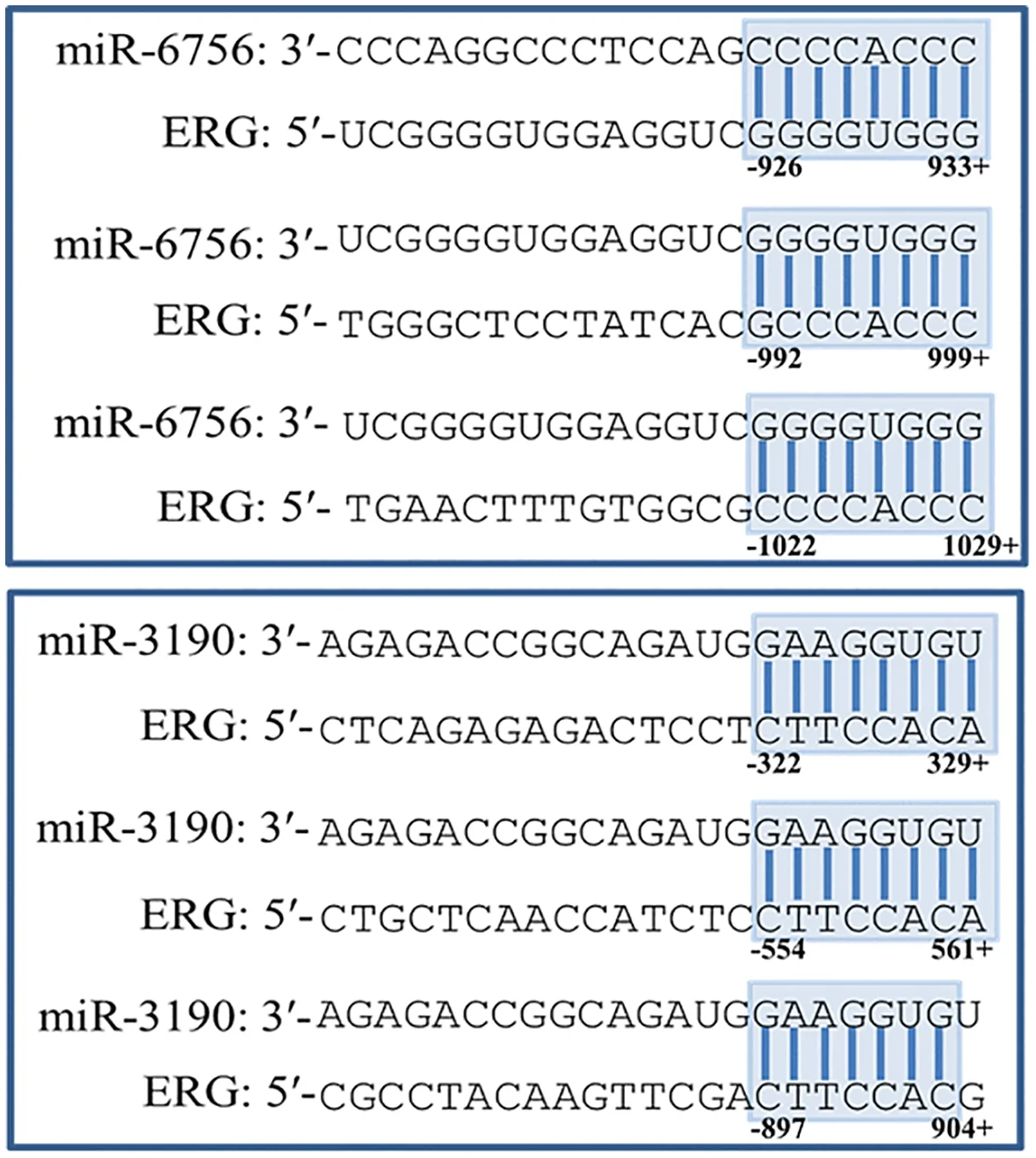
The binding sites of miR-6756 (−926 + , −992 + , −1022+) and miR- 3190 (−322 + , −554 + , −897+) on ERG mRNA.

Predicting miRNAs and their targets is not easy since only a short stretch of about seven nucleotides has to match the sequence of a target mRNA, while about 18 nucleotides (the minimum length of PCR primers) would be necessary to provide statistical specificity for a unique binding site. Therefore, there might be dozens of miRNAs binding the same target and vice versa, one species of mRNA may be targeted by a multitude of miRNAs. Various computational methods have been developed to predict miRNAs and their targets leading to the establishment of a substantial variety of miRNA databases. To identify candidate miRNAs for ERG, we performed an analysis on *miRDB*. *miRDB* revealed that the ERG mRNA is a target of miR-6756 and miR-3190 and contains three potential binding sites for each miRNA with high scores (99) (Fig 1). Therefore, we proceed to test this binding in various experimental conditions.

### miR-6756 and miR-3190 reduce ERG mRNA and protein expression

**Fig 2:**
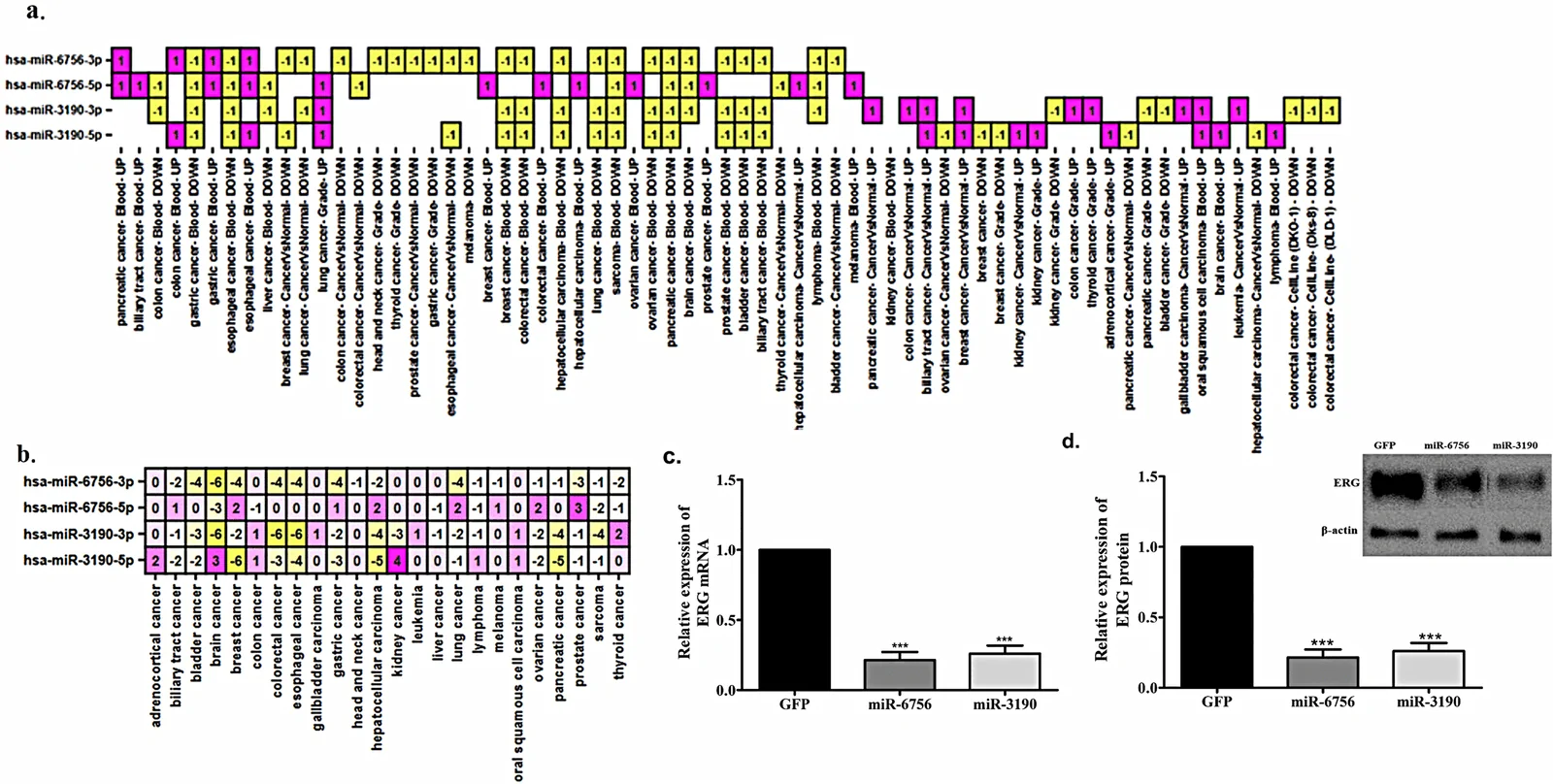
Bioinformatic profiling of hsa-miR-6756 and hsa-miR-3190 expression across human cancers. (a, b) Expression patterns of hsa-miR-6756-3p, hsa-miR-6756-5p, hsa-miR-3190-3p, and hsa-miR-3190-5p across various cancer types, aggregated from public transcriptomic datasets. Expression values are represented as log_2_ fold-change, where positive values (pink) indicate upregulation in and negative values (yellow) indicate downregulation. White indicates no significant change. miR-6756 and miR-3190 transfection in HUVECs. (c) ERG mRNA expression relative to GAPDH by qPCR; (d) ERG Protein expression relative to β-actin by western blotting and its quantification. Data expressed as mean  ±  SD, ***p  <  0.001.

Our study was initiated by a bioinformatic analysis revealing that hsa-miR-6756 and hsa-miR-3190 are frequently dysregulated across a broad spectrum of human malignancies, with a notable pattern of downregulation in solid tumor tissues (Fig 2a and 2b). To explore their potential functional role in a cancer-relevant pathways, we hypothesized “miR-6756 and miR-3190 might modulate ERG, a transcriptional oncogene critical for endothelial cell function that regulate the angiogenic signaling”. We therefore tested this hypothesis in human umbilical vein endothelial cells (HUVECs), a canonical model for angiogenic studies. To study the expression of ERG at mRNA and protein levels the qPCR and western blotting were performed respectively, after transfection with miR-3190 and miR-6756. The results showed that the expression was significantly reduced at both the mRNA (*p <  0.001*) and protein levels (*p  <  0.001*) after transfections with miR-6756 and miR-3190 in comparison with CMV-eGFP transfected cells as shown in Fig 2c and 2d. This confirms that both miRNAs are potent suppressors of ERG in endothelial cells, providing a direct mechanistic link between their bioinformatically observed loss in cancers and a potential disruption of angiogenic regulation.

### ERG mRNA is the direct target of miR-6756 and miR-3190

**Fig 3:**
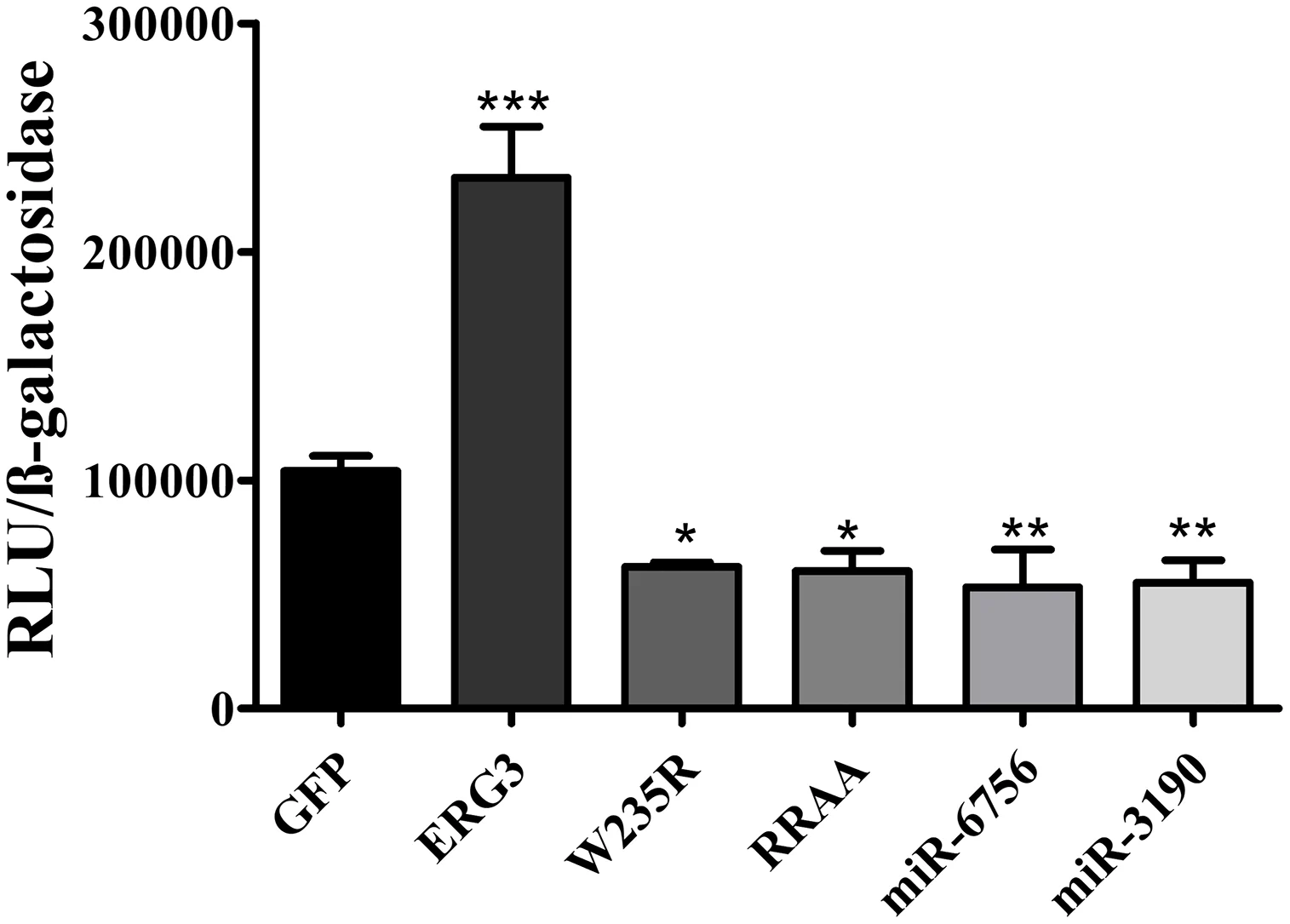
Reporter gene assay in HEK293 cells to determine the transcriptional activity of ERG. ERG NanoLuc vector coexpressed with ERG3, miR-6756 and miR-3190 in three independent experiments (n = 3). Data expressed as mean  ±  SD, *p  <  0.05, **p  <  0.01, ***p  <  0.001. GFP = Green Fluorescent Protein, W235R = ERG mutant where tryptophan at position 235 is replaced with arginine, RRAA = ERG mutant where two arginine residues substituted by alanine.

To elucidate the effect of the binding of miR-6756 and miR-3190 on the ERG mRNA and transcription activity of ERG, a NanoLuc reporter assay was performed. The ERG NanoLuc vector was co-expressed with miR-6756 and miR-3190 independently in HEK293 cells. In HEK293 cells significantly reduced transcription activity was observed in the cells transfected with miR-6756 (*p* < *0.001*) and miR-3190 (*p < 0.001*) in comparison with the control and ERG transfected cells. The ERG mutants (RRAA and W235R) were also used as control and showed reduced transcription activity (Fig 3).

### miR-6756 and miR-3190 affect the endothelial cell migration and proliferation

**Fig 4:**
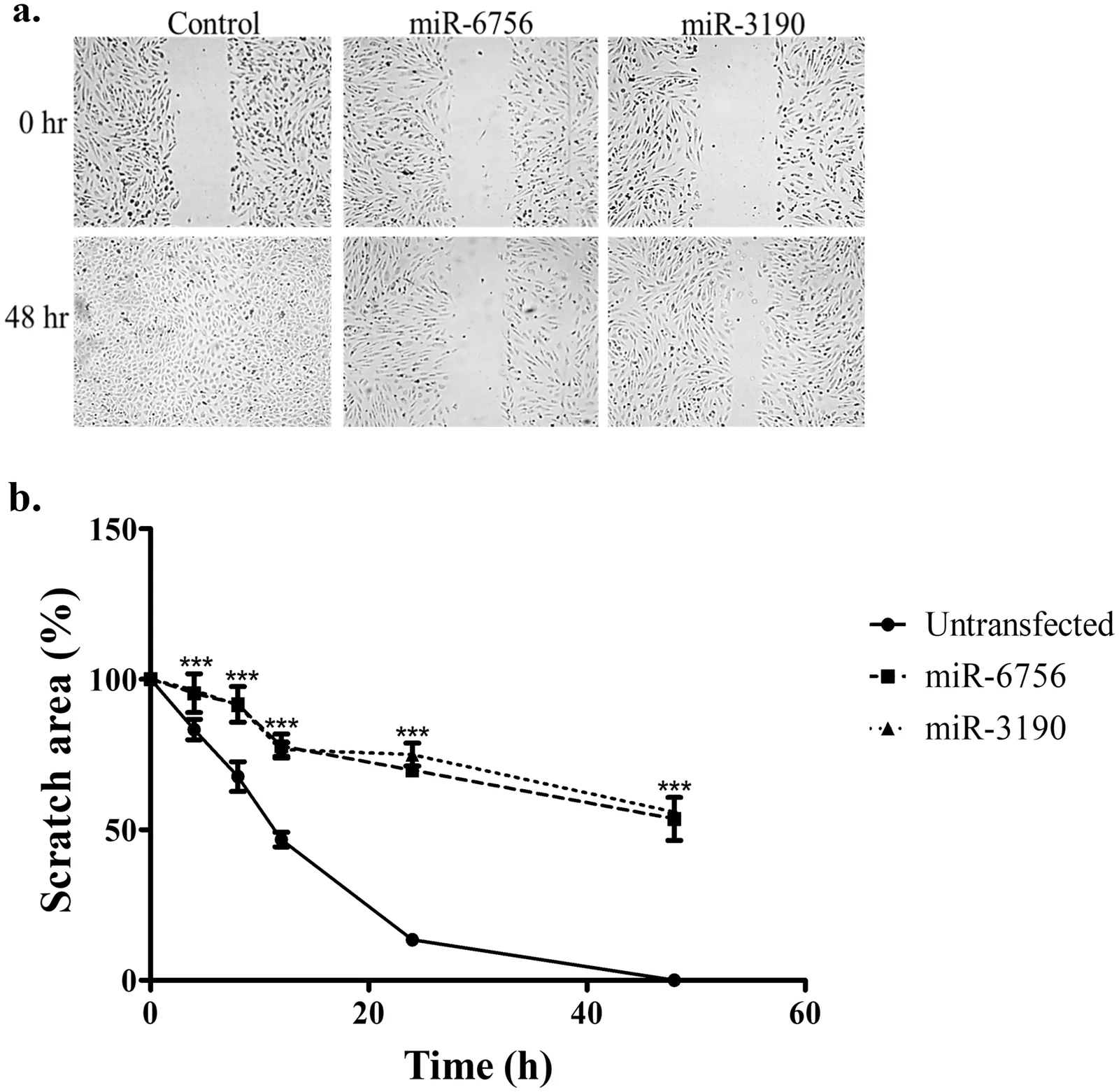
miR-6756 and miR-3190 inhibit the migration of HUVECs in three independent experiments. Data expressed as percentage of area migrated. a) Representative images; b) Percentage rate of migration; Data expressed as mean  ±  SD, ***p  <  0.001.

To elucidate the effects of miR-6756 and miR-3190 mediated ERG suppression on cell migration and proliferation scratch assay was performed. HUVECs were separately transfected with CMV-eGFP, miR-6756, and miR-3190. After the transfections when the monolayer of cells has formed a scratch was made and the migration of the cells was evaluated at the interval of 4 hours to 48 hours. HUVECs transfected with miR-6756 and miR-3190 showed reduced migration rates in comparison to CMV-eGFP transfected cells. In CMV-eGFP transfected cells scratch closure was observed within 48 hours while in miR-6756 and miR-3190 transfected cells the gap was visible. The area migrated by the cells was measured and demonstrated in terms of percentage as shown in Fig 4.

### miR-6756 and miR-3190 mediated regulation of ERG targets

**Fig 5:**
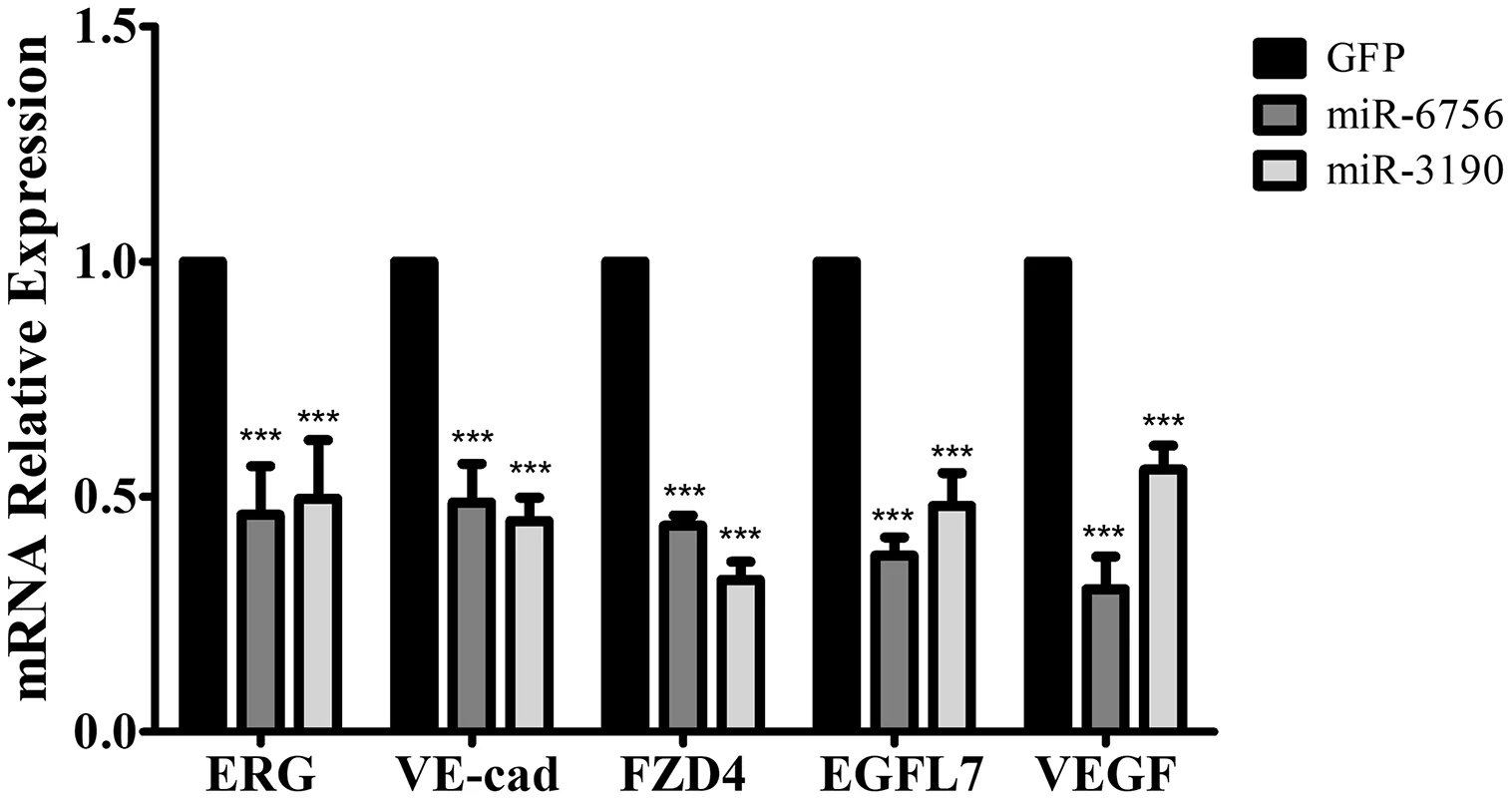
mRNA expression of ERG angiogenic targets after transfection of HUVECs with miR-6756 and miR-3190; Data expressed as mean  ±  SD, ***p  <  0.001.

To elucidate whether miR-6756 and miR-3190 regulate ERG downstream angiogenic targets, the mRNA expression of ERG downstream target genes was analyzed. It was observed that the downstream target genes (*VE-cadherin, FZD4, EGFL7,* and *VEGF*) of ERG involved in angiogenesis were significantly reduced after transfections of HUVECs with miR-6756 and miR-3190 in comparison to control cells (Fig 5). GAPDH was used to normalize the data.

### miR-6756 and miR-3190 mediated ERG down-regulation affect the VE-cad expression and promoter activity

**Fig 6:**
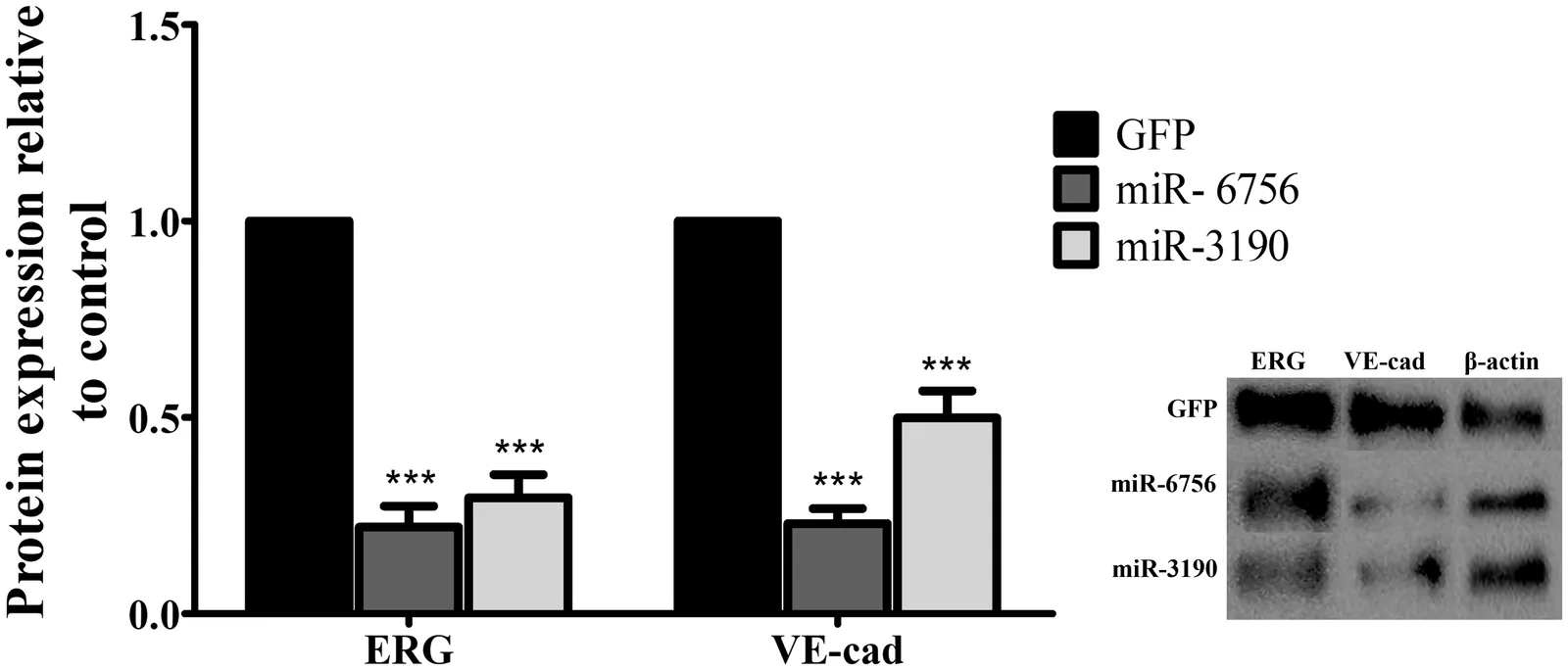
miR-6756 and miR-3190 influence VE-cad protein expression. Western blotting was done using anti-VE-cad antibody. β-actin was used as a loading control. Quantiﬁcation of ERG and VE-cad by ImageJ. Expression was normalized to the β-actin signal of the respective sample ***p  <  0.001.

**Fig 7:**
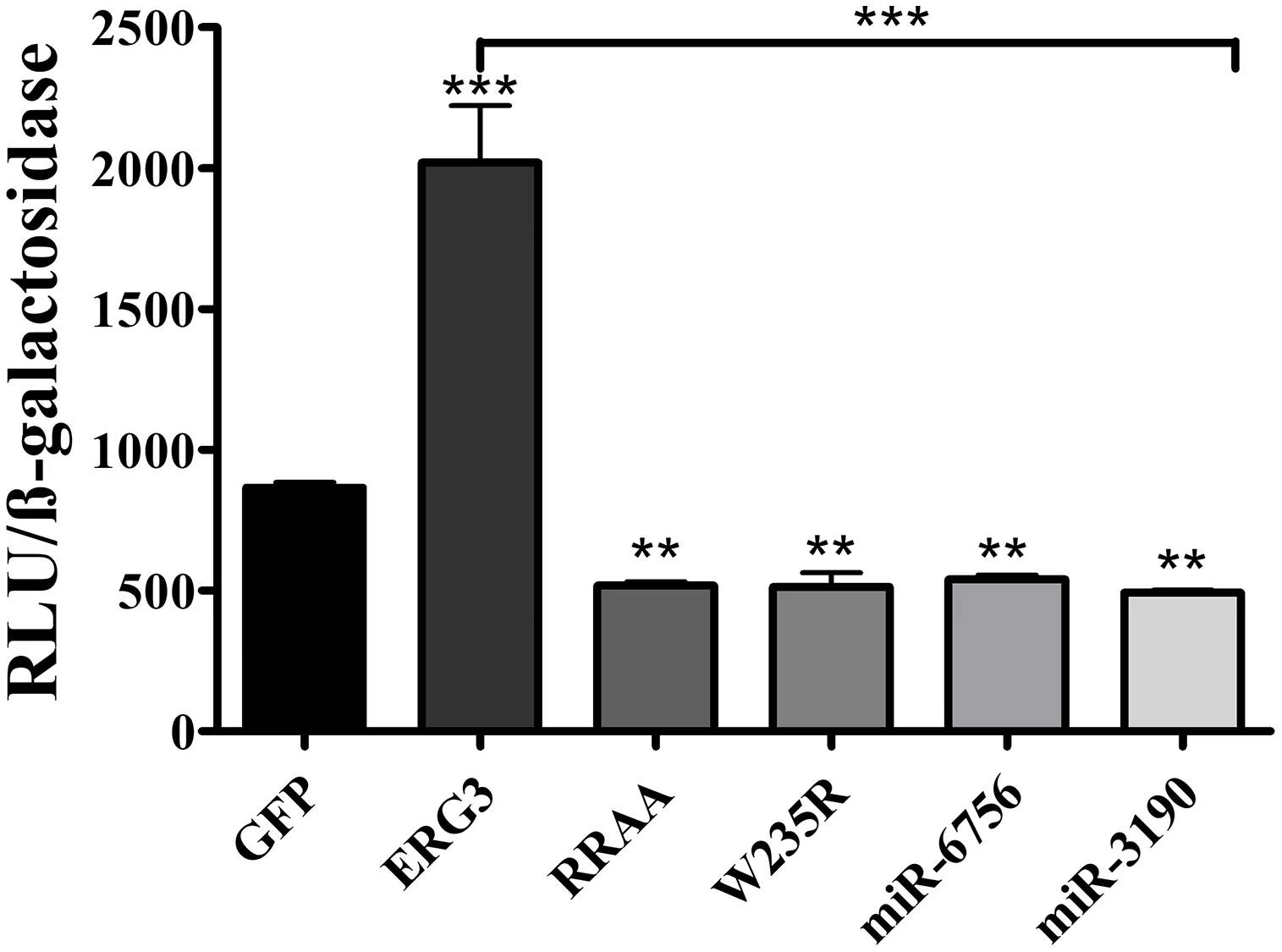
Reporter gene assay using VE-cad promoter in HUVECs after co-expression of miR-6756 and miR-3190, a constitutively expressing β-galactosidase was used for normalization. n = 3. Data expressed as mean  ±  SD, **p  <  0.01, *** p  <  0.001.

The protein expression of VE-cad was also reduced significantly in response to miR-6756 and miR-3190 overexpression (Fig 6). To demonstrate if the *VE-cad* promoter activity is influenced by miR-6756 and miR-3190 mediated ERG down-regulation, a reporter gene assay was carried out. The *VE-cad* promoter (pGL3-VE-Cad-Luc; courtesy from Peter Huber) region (−2486, + 24) was co-transfected with miR-6756 and miR-3190 in HUVECs. Appropriate controls, i.e., ERG and ERG mutants (W235R and RRAA) were also used in the experiment. The results showed that miR-6756 and miR-3190 significantly reduced the activity of the *VE-cad* promoter implying that the ERG angiogenic targets are also influenced by miR-6756 and miR-3190 as shown in Fig 7.

## Discussion

AngiomiRs are a class of miRNAs, which are involved in the regulation of angiogenic signaling pathways. Pro-angiomiRs stimulate the process of angiogenesis by targeting negative regulators of angiogenesis, while anti-angiomiRs inhibit this biological process by targeting positive regulators [32]. It is known that single miRNAs can target many different mRNAs, which often occurs cooperatively with other miRNAs. In this way, complex gene regulatory networks can be modulated [33]. The transcription factor ERG is expressed throughout life in the endothelium and regulates multiple pathways involved in vascular homeostasis and angiogenesis [34]. In our study, we explored miR-6756 and miR-3190 for a potential anti-angiogenic potential by targeting ERG.

Our results indicated that the ERG mRNA is a potential target of miR-6756 and miR-3190, as identified by bioinformatics analysis. We tested this hypothesis experimentally by co-expression of miR-6756 and miR-3190 with an ERG reporter, which revealed a decrease in reporter gene activity as compared to controls. We assume that miR-6756 and miR-3190 binding to the seed sequence on the ERG mRNA might inhibit the translation of ERG, alternatively the stability of the ERG mRNA. Consistent with the reporter assays the exogenous miR-6756 and miR-3190 decreased the expression of ERG at mRNA and protein levels in HUVECs *in-vitro*; in line with the notion that ERG expression is reduced by miR-6756 and miR-3190. Furthermore, scratch assays implied an inhibitory effect of miR-6756 and miR-3190 transfected into endothelial cells. The expression of ERG downstream targets involved in angiogenesis was also significantly decreased, suggesting a negative impact of miR-6756 and miR-3190 on angiogenic mediator ERG. Finally, VE-cad an established ERG target regulating angiogenesis was found to decrease significantly after transfection of miR-3912, which was further corroborated by reporter assays using a VE-cad promotor construct with known ERG and miR-6756 and miR-3190 binding sites. These results suggest that miR-6756 and miR-3190 may act as anti-angiomiR by influencing ERG expression in the endothelium.

The possible degradation of ERG mRNA at the posttranscriptional level and inhibition of protein translation by miR-6756 and miR-3190 may provide new targets for the regulation of angiogenesis. A similar observation was made by Fang et al., 2019 [35] where they showed that miR-622 downregulated the angiogenic factor VEGFA in colorectal cancer. Other miRNAs targeting ERG such as miR-196a and miR-196b, caused the downregulation of ERG in myeloid cells and T lymphoblastic cells [36]. Our findings suggested a potential role for the downregulation of miR-6756 and miR-3190 in modulating endothelial ERG expression, which may have a downstream influence on angiogenesis in these malignancies. ERG regulates various endothelial genes like VE-cad [15], VEGF [37], FZD4 [16], and EGFL7 [38], which are involved in angiogenesis, cell adhesion, as well as vascular and junction stability. The expression of these genes was also downregulated in our study in response to miR-6756 and miR-3190 overexpression, thus supporting its role in regulating angiogenesis via ERG. VE-cad maintains endothelial cell survival by stabilizing the intercellular junctions [15]. Inhibition of ERG results in a significant decrease in VE-cad, which may modulate the angiogenic signals. Identification of inhibitors, which can suppress ERG expression and in return VE-cad can be seen as therapeutic candidates specifically in situations of pathological angiogenesis as in cancer.

Many studies have indicated that miRNAs can be used for therapeutic purpose by regulating genes involved in angiogenesis and therefore also in tumor progression [39–41]. Previously, several miRNAs like miR-221, miR-222 [42], miR-16, miR-424 [43], miR-1271 [44], and miR-145 [45,46] have been shown to have the ability to regulate the cell migration and angiogenic signaling. The identification of miR-6756 and miR-3190 is significant in this regard as they present another angiogenic target in pathological conditions.

## Conclusion

This study identifies that miR-6756 and miR-3190 are regulators of the angiogenic transcription factor ERG, demonstrating that their upregulation suppresses endothelial cell behavior and downstream vascular functions regulated by ERG. Thus, targeting the miR-6756/miR-3190/ERG axis may represent a novel strategy to interfere with various pathological forms involving angiogenesis. However, these translational insights are limited by reliance on *in-vitro* models. Further, *in-vivo* studies can help to identify miR-6756 and miR-3190 as potential anti-angiogenic targets.

## Supporting information

S1 Data Raw data graphs. (ZIP)

S1 File Supporting blot/gel data. (PDF)
