# Supplementary material for: Modulation of angiogenic transcription factor ERG via miR-6756 and miR-3190
Source: PLoS One. 2026 Sep 25;21(9):e0358739. doi: 10.1371/journal.pone.0358739 (PMC13614596; doi:10.1371/journal.pone.0358739)
Supplement: Supplementary file 1 [file pone.0358739.s002.pdf]

Supplementary file

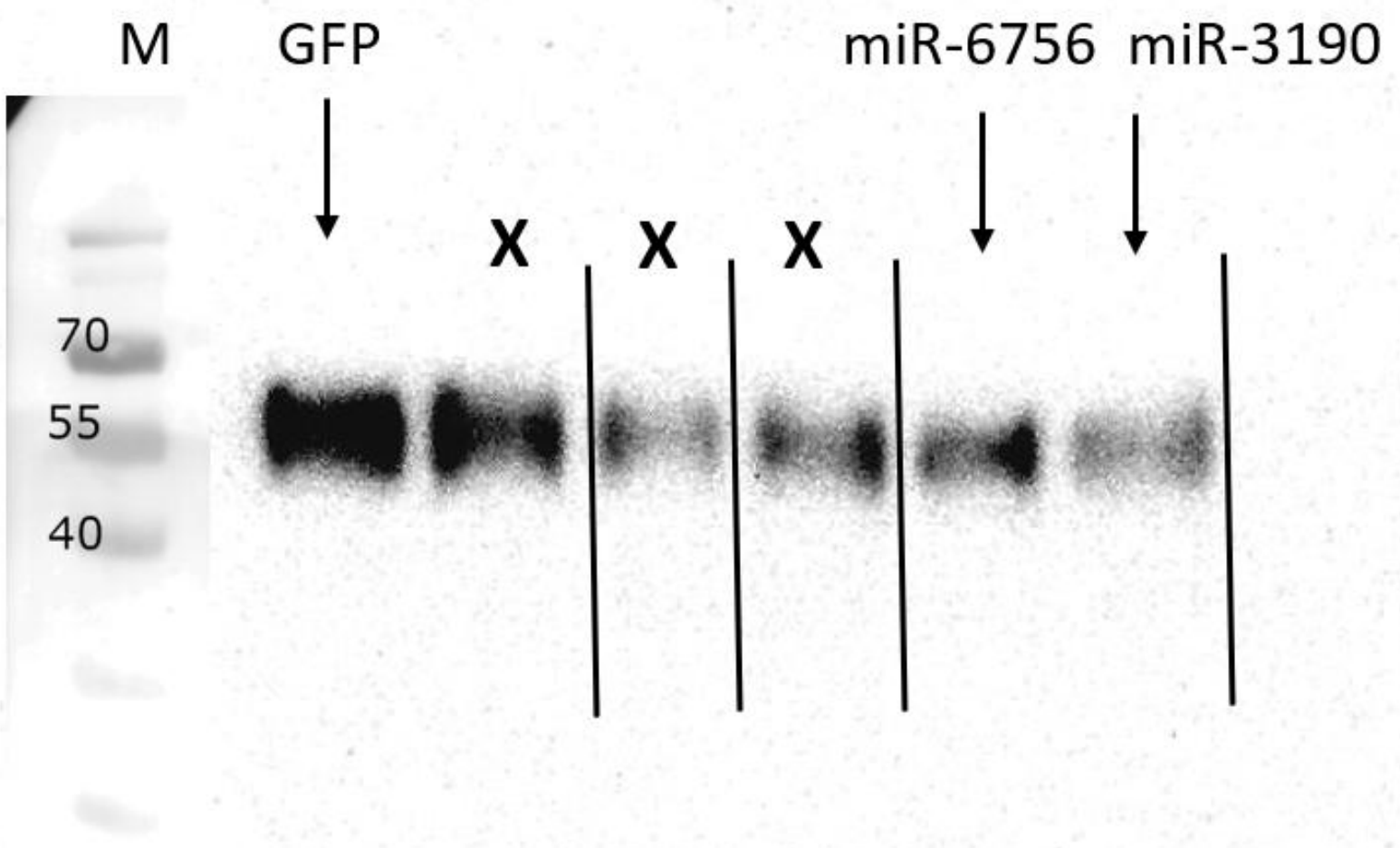

ERG for figure 2d and figure 6

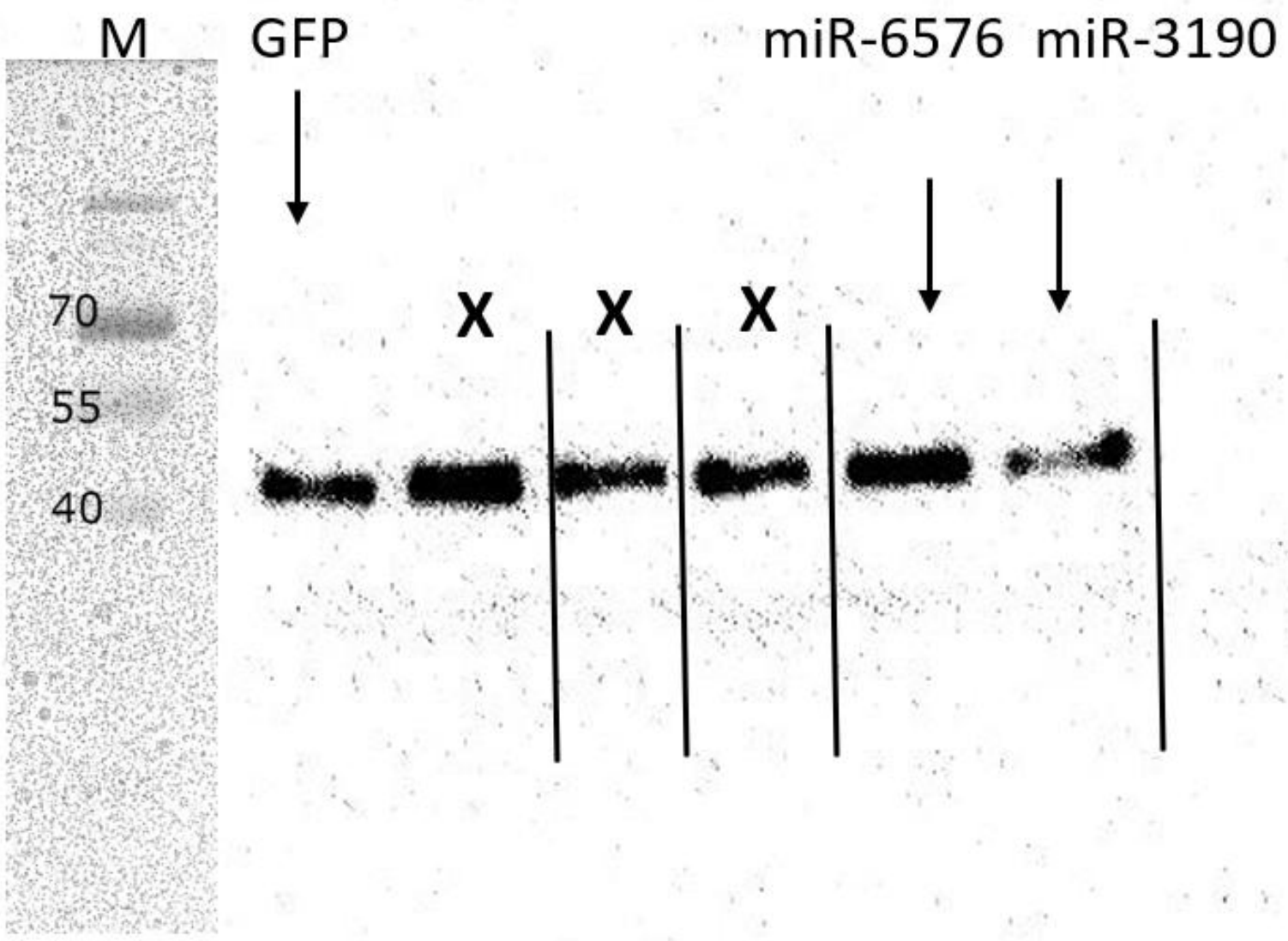

Actin control for figure 2d and figure 6

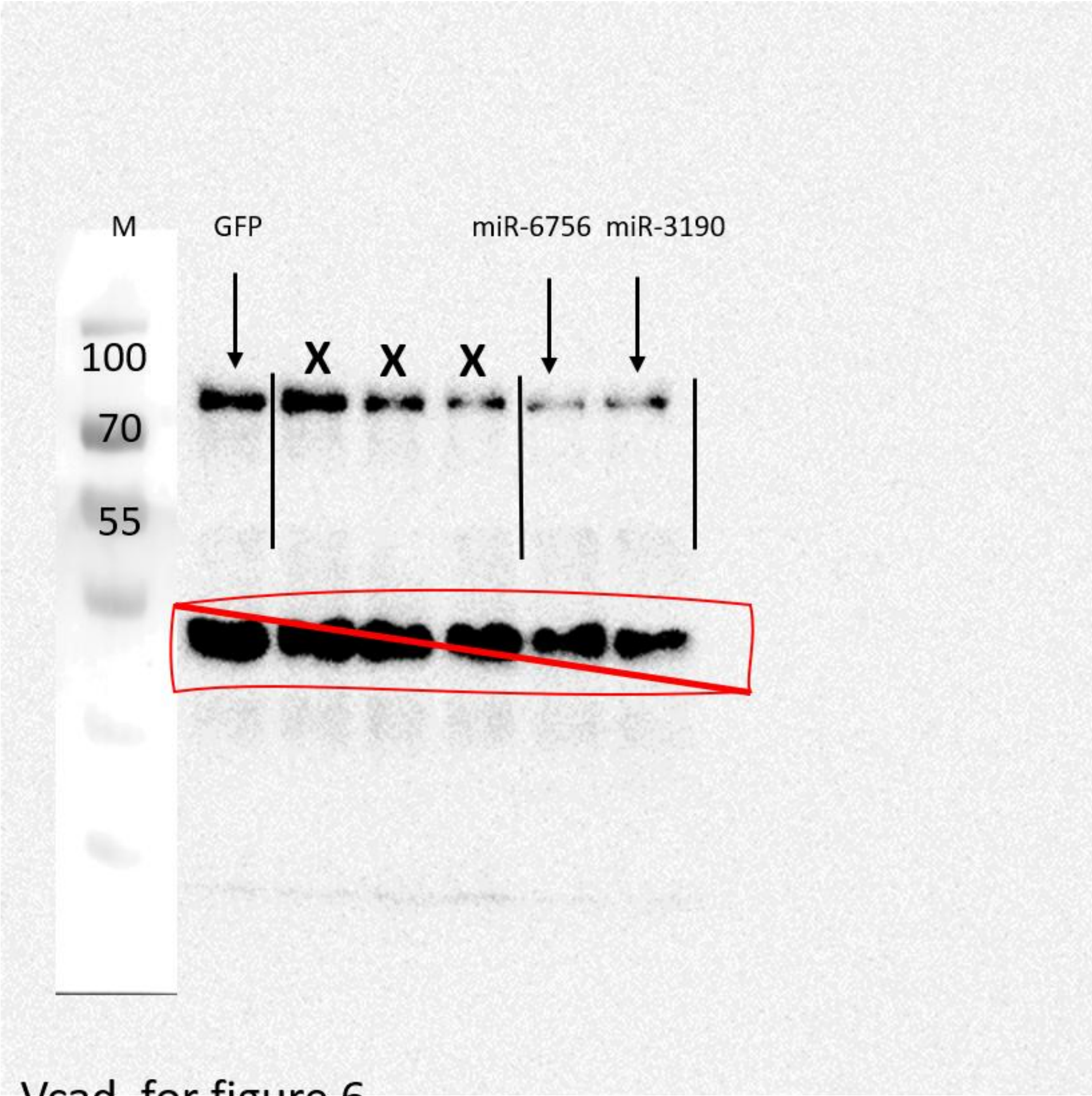

Vcad for figure 6
